# Voltage effects on muscarinic acetylcholine receptor‐mediated contractions of airway smooth muscle

**DOI:** 10.14814/phy2.13856

**Published:** 2018-09-05

**Authors:** Iurii Semenov, Robert Brenner

**Affiliations:** ^1^ Frank Reidy Research Center for Bioelectrics Old Dominion University Norfolk Virginia; ^2^ Department of Cell and Integrative Physiology University of Texas Health Science Center San Antonio San Antonio Texas

**Keywords:** Acetylcholine receptors, airway smooth muscle, calcium channels, contractions, voltage

## Abstract

Studies have shown that the activity of muscarinic receptors and their affinity to agonists are sensitive to membrane potential. It was reported that in airway smooth muscle (ASM) depolarization evoked by high K^+^ solution increases contractility through direct effects on M3 muscarinic receptors. In this study, we assessed the physiological relevance of voltage sensitivity of muscarinic receptors on ASM contractility. Our findings reveal that depolarization by high K^+^ solution induces contraction in intact mouse trachea predominantly through activation of acetylcholine release from embedded nerves, and to a lesser extent by direct effects on M3 receptors. We therefore devised a pharmacological approach to depolarize tissue to various extents in an organ bath preparation, and isolate contraction due exclusively to ASM muscarinic receptors within range of physiological voltages. Our results indicate that unliganded muscarinic receptors do not contribute to contraction regardless of voltage. Utilizing low K^+^ solution to hyperpolarize membrane potentials during contractions had no effect on liganded muscarinic receptor‐evoked contractions, although it eliminated the contribution of voltage‐gated calcium channels. However, we found that muscarinic signaling was potentiated by at least 42% at depolarizing voltages (average −12 mV) induced by high K^+^ solution (20 mmol/L K^+^). In summary, we conclude that contractions evoked by direct activation of muscarinic receptors have negligible sensitivity to physiological voltages. However, contraction activated by cholinergic stimulation can be potentiated by membrane potentials occurring beyond the physiological range of ASM.

## Introduction

Airway smooth muscle (ASM) contraction is primarily mediated by so called “pharmaco‐mechanical coupling” via activation of M2 and M3 muscarinic cholinergic receptors linked to G_i/o_‐ and G_q_‐coupled receptors, respectively (Eglen et al. 1996; Hall 2000; Ehlert 2003; Sanderson et al. 2008). Stimulation of M3/G_q_‐coupled receptors activates phospholipase C which cleaves PIP_2_ and triggers Ca^2+^ release from sarcoplasmic reticulum (SR) via IP_3_ receptors to initiate contraction (Berridge 1993). M2/G_i/o_ receptor activation contributes to contraction through inhibition of cAMP signaling (Fryer and Jacoby 1998). Muscarinic receptor activation also causes a distinct mode of contraction through activation of ionic currents that depolarize the plasmallema (Benham et al. 1985; Lee et al. 1993), so called “electro‐mechanical coupling”. Depolarization gates opening of voltage‐dependent calcium channels that mediate calcium influx and contribute to contraction (Somlyo and Somlyo 1968). However, the pharmacomechanical and electromechanical coupling mechanisms have been blurred by studies showing voltage sensitivity of numerous G protein–coupled receptors (Mahaut‐Smith et al. 2008). Among these, the predominant G protein–coupled receptors in ASM, the M2 and M3 muscarinic receptors, have been shown to be directly sensitive to voltage (Mahaut‐Smith et al. 2008; Rinne et al. 2015). These reports suggest that when acetylcholine or carbachol is used as an agonist, M2 receptor is activated by depolarization, while M3 receptor is inhibited by depolarization (Mahaut‐Smith et al. 2008; Rinne et al. 2015).

Previously, it was found that chemical depolarization with 60 mmol/L K^+^ extracellular solution increased ASM contractile force in a manner that is directly dependent on M3 receptor signaling (Liu et al. 2009). An important question is whether physiologically relevant voltage changes also increase muscarinic‐evoked contractions of ASM. While chemical depolarization (e.g., using 60 mmol/L K^+^ extracellular solution) is a convenient method to assess the effect of voltage on contraction of muscle preparations, the actual effect on tissues can be complex to interpret. ASM generally traverse modest voltage ranges following cholinergic receptor activation, with reports indicating voltages that are not more positive than −30 mV (Janssen 1998; Semenov et al. 2011). In contrast, 60 mmol/L K^+^ extracellular solution can theoretically drive voltage to more depolarized potentials. One can estimate a Nernst potential of −21 mV, assuming a predominant permeability to potassium and an expected 140 mmol/L intracellular potassium concentration. Actual measurements suggest depolarizations to ~0 mV with 50 mmol/L potassium solution (Imaizumi and Watanabe 1981). Furthermore, high K^+^ also depolarizes parasympathetic nerves embedded in ASM preparations, possibly contributing to contractions via nerve‐mediated acetylcholine release (Alberts et al. 1982). This is a particular issue for ASM, since a superficial buffer barrier to calcium influx is believed to bias contraction responses to cholinergic receptors more so than voltage‐gated calcium channels (Janssen et al. 1999).

The aim of this study is to assess the contribution of physiologically relevant depolarization‐driven activation of muscarinic receptors on ASM contractility in intact tissues. We hypothesize that contractions in high K^+^ solutions are confounded by depolarization of ASM beyond their physiological range, and also confounded by effects of depolarization via activation of embedded nerves. Both of these confounds may account for an overestimation of the physiological role of depolarization on muscarinic receptor signaling in ASM (Liu et al. 2009). To test the hypothesis, we took pharmacological approaches to isolate effects of chemical depolarization (high K^+^) on muscarinic receptor‐dependent contractions. Our findings reveal that chemical depolarization of intact trachea preparations predominantly mediate contractions through acetylcholine release from embedded nerves, and to a lesser extent by depolarization of the smooth muscle cells. Furthermore, voltage‐dependent calcium channel blocker, nifedipine, or removal of extracellular calcium abolished contraction via direct voltage‐dependent calcium influx. By eliminating these aforementioned signaling pathways, we find that depolarization have effects on muscarinic‐evoked contractions but outside the physiological range of ASM voltages.

## Materials and Methods

### Tissue preparations and contraction recordings

The methods were similar to those described previously (Semenov et al. 2011). Animals used in these studies were the background C57BL/6J mice strain from Jackson Labs. All animal procedures were reviewed and approved by the University of Texas Health Science Center at San Antonio Institutional Animal Care and Use Committee. For tracheal contraction studies, animals were first deeply anesthetized with isoflurane in an induction chamber and then sacrificed by cervical dislocation. Trachea were quickly removed and dissected clean of surrounding tissues in ice‐cold normal physiological saline solution (PSS). The tracheal tube was cut below the pharynx and above the primary bronchus bifurcation. Two metal wires, attached to a force transducer and micrometer (Radnoti, LLC), were threaded into the lumen of the trachea. The trachea was placed into an organ bath oxygenated by O_2_–CO_2_ mixture (95% O_2_ and 5% CO_2_), at 37°C. This preparation was not denuded of epithelial cells.

Two platinum electrodes placed on the sides of the preparation were used to deliver electrical field stimulation (EFS). The electrodes were positioned 4 mm apart and this distance was maintained constant throughout the study. All wires and soldered connections were sealed with Sylgard (Sylgard 184 Silicone Elastomer, Dow Corning Corp., Midland MI) to insulate from the bathing solution and to prevent leaching of deleterious metals into the tissue bath. Dependencies of contraction force on stimulation frequency and voltage were obtained and analyzed. Stimulation at 30 Hz by 0.5 msec pulses (44 V) was found to be an optimal for our experimental setup to achieve reproducible (no tissue damage) near‐maximal contractile responses.

Resting tension of ASM preparation was continuously readjusted in the water bath to 1 g for 1 h. Then, the preparation was challenged with 60 mmol/L K^+^ PSS twice or more until reproducible contraction responses were achieved. Responses to the subsequent experimental challenges with drugs were normalized to the maximal contraction response in the 60 mmol/L K^+^ PSS solution. For experiments that involved two or more cholinergic challenges, we found that the responses to subsequent challenges did not show significant fatigue (*P* = 0.19, *n* = 9, students paired *t* test). On average, the next challenge produced 0.95 ± 1% reduction in response from that of the previous challenge. In case of six challenges separated by 10 min’ intervals, sixth challenge produced 2 ± 1% reduction in response in comparison with the first. Normal PSS used was (mmol/L): 119 NaCl, 4.7 KCl, 2.0 CaCl_2_, 1.0 KH_2_PO_4_, 1.17 MgSO_4_, 18 NaHCO_3_, 0.026 EDTA, 11 glucose, and 12.5 sucrose. The pH of the solution was adjusted to 7.3 by 95% O_2_–5% CO_2_ mixture. Normal PSS had a total 5.7 mmol/L K^+^ derived from 4.7 mmol/L KCl plus 1.0 mmol/L KH_2_PO_4_. “Low” K^+^ PSS solution was used to measure contractions in relatively hyperpolarized conditions. Low K^+^ PSS consisted of 1.0 mmol/L potassium derived from the KH_2_PO_4_ with no added KCl. To maintain proper osmolality both 20 mmol/L and 60 mmol/L potassium PSS contained reduced sodium of 103.7 and 63.7 mmol/L, respectively. In 0 Ca^2+^ solutions, CaCl_2_ was replaced with 100 *μ*mol/L EGTA to remove residual Ca^2+^ ions.

Botulinum neurotoxin A (BTX) treatment protocol was adopted from Moffatt et al. (2004) with the few modifications. Briefly, BTX stock solution was added to tracheas submerged in DMEM to achieve final concentration of BTX 50 nmol/L. Tracheas were incubated with BTX for 3 h in a tissue culture incubator at 37°C. Tracheas for control experiment were incubated for the same period at the same conditions with the equal volume of vehicle instead. After incubation, tracheas were mounted in the organ bathes as described above and rinsed with normal PSS. Resting tension was readjusted for 1 g continuously during 30 min. Then, preparations were challenged with 60 mmol/L potassium PSS twice. MacLab 8 A/D data‐gathering system (AD Instruments, Inc., Colorado Springs, CO) installed on Macintosh computer was used to visualize the signals during acquisition and later during data analysis.

### Tracheal smooth muscle cell isolation and sharp electrode recordings

To find detailed procedures for tracheal smooth muscle cells isolation and sharp electrode recordings, please refer to Semenov et al. (2011). Here, only a brief description is provided. The dorsal muscle layer of trachea was minced into the small pieces in Ca^2+^‐free Krebs solution at the presence of 2.5 U/mL of papain (MP Biomedicals, Solon, OH, USA), 1 mg/mL of BSA fraction V. Then, muscle pieces were shaken at 37°C (250 moves/min) on a rocking platform in the same solution for 20 min, rinsed in Ca^2+^‐free Krebs solution, mixed with 12.5 U/mL of type VII collagenase (Sigma‐Aldrich Corp.), and placed back on the rocking platform for 10 min. Digested pieces of tissue were washed in Ca^2+^‐free Krebs‐BSA solution and triturated with a fire polished Pasteur pipette to dissociate single tracheal myocytes. Isolated cells were stored on ice in Ca^2+^‐free Krebs‐BSA solution to be used within 6 h time. Drop (50 *μ*L) of the suspension of isolated cells was placed in an open 1.0 mL perfusion chamber mounted on the stage of an inverted microscope. Cells were allowed to adhere to the glass bottom of the chamber and then were continuously perfused with the PSS (0.5–1 mL/min) containing 5.7, 1.0, or 20 mmol/L of K^+^ ions. pH of the solution was maintained at 7.35 by oxygenation with 95% O_2_–5% CO_2_ mixture and temperature was held at 37°C using an automatic temperature controller (TC‐324B, Warner Instruments, LLC, Hamden, CT, USA).

Membrane potentials (*V*_m_) of the single cells were measured using the sharp electrode technique with an EPC‐9 amplifier (Heka Electronics). Sharp pipettes (20–60 MΩ) were pulled from borosilicate capillary glass (1B150F‐4, WPI) using a Sutter P‐87 pipette puller. Pipettes were filled with the 2 mol/L KCl solution. One percentage of Agarose Type IX‐A was added to prevent leak of the pipette solution into the cell. Impalement of cell by a pipette was recognized as a sudden drop of voltage. Resting membrane potentials were recorded for 1 min to ensure a stability of the baseline. “Igor” software (Igor, WaveMetrics Inc.) was used for statistical analyses. Significance was determined with Student's *t* test for paired or unpaired data as appropriate. The effects were deemed significant when a *P* < 0.05 was obtained. The results are presented as the means ± standard error of the mean where applicable.

## Results

### Trachea contraction evoked by chemical depolarization consists of two major parts: atropine‐sensitive and nifedipine‐sensitive components

**Figure 1:**
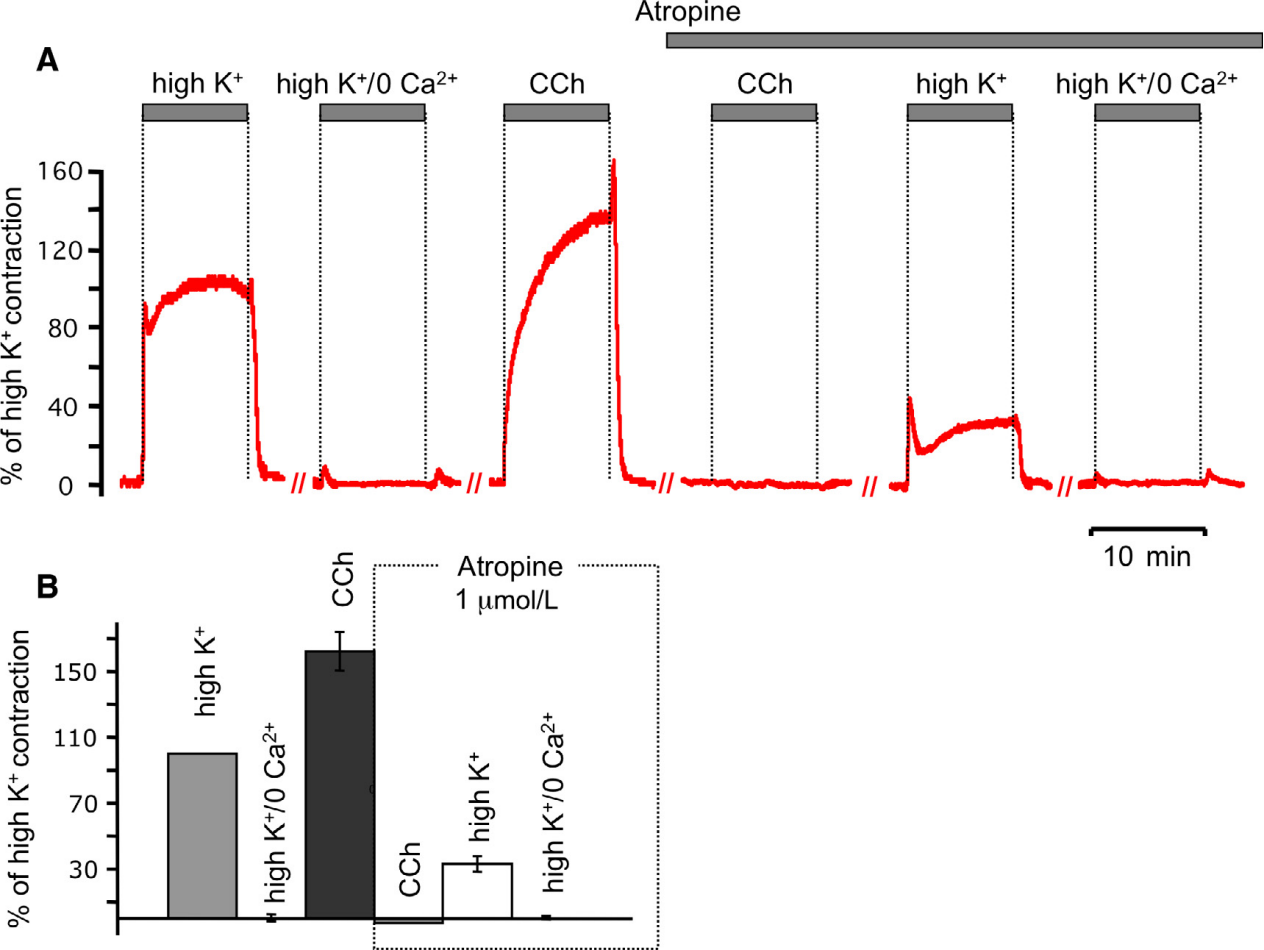
Trachea contraction evoked by chemical depolarization consists of a larger atropine‐sensitive and smaller, atropine‐insensitive component. (A) Treatments are indicated above the representative traces of ASM contraction: high K^+^ (60 mmol/L KCl and 2 mmol/L CaCl_2_), high K^+^/0 Ca^2+^ solution (60 mmol/L KCl, 0 mmol/L CaCl_2_, and 100 *μ*mol/L EGTA), and CCh (0.5 *μ*mol/L carbachol) in control and after antagonism of muscarinic receptors with Atropine (1 *μ*mol/L). All traces are normalized to contractions induced in high K^+^ solution. Hashed lines (//) indicate a 20 min wash with normal physiological saline solution. (B) Summarized data for A. Amplitude was estimated after contractions reached at the plateau phase. Results are expressed as the means ± standard error of the mean. For any bar *n* = 6.

To understand the role of voltage on muscarinic signaling, we wished to identify an experimental approach to affect voltage in ASM and measure contractions due solely to muscarinic receptor signaling. Figure 1A demonstrates a tracheal ring contraction using a depolarizing high K^+^ (60 mmol/L) solution, and examines the contribution of muscarinic receptors (atropine‐sensitive contractions) and other pathways (atropine‐insensitive contractions). High K^+^ in this preparation yielded approximately 60% of the contraction from a half‐maximal concentration of cholinergic agonist carbachol (0.5 *μ*mol/L, Fig. 1A, summarized in 1B). Atropine, a muscarinic acetylcholine receptor antagonist, reduced the contractile response significantly and revealed an atropine‐insensitive component of high K^+^ contraction (Fig. 1A, right). The atropine‐insensitive component has a reduced amplitude and prominent two‐phase appearance (Fig. 1, A): (1) distinct transient peak (46 ± 5% of maximal contraction response in the 60 mmol/L KCl at control conditions) and (2) smaller sustained plateau (32 ± 2%, *n* = 6). Zero external calcium eliminated the atropine‐insensitive component of contraction, which was unsurprising given that this likely represents contraction mediated by Ca^2+^ influx via voltage‐gated calcium channels. However, we were surprised to find that the Atropine‐sensitive component of contraction was also eliminated by 0 external calcium (Fig. 1A, left). This is paradoxical given that M3 muscarinic receptors primarily mediate contraction through calcium‐store release mechanisms, and a transient contraction is expected. These results indicate that high K^+^ contraction is largely dependent on calcium influx, of which a majority is atropine sensitive, and a minority is independent of muscarinic receptors.

### The atropine‐sensitive component of high K^+^ contraction depends on acetylcholine release from the nerve terminals

As was shown above, the majority of the contractile response initiated by high K^+^ depends on two events: (1) Ca^2+^ influx and (2) muscarinic receptor activation. A hypothesis to explain these would be that high K^+^ depolarizes and activates cholinergic neurotransmission, which would be dependent on calcium influx into nerve endings and release of acetycholine onto apposing muscle. We tested this possibility by using botulinum toxin A (BTX) to block acetylcholine release from nerve terminals. BTX acts as a zinc‐dependent protease that effectively prevents exocytosis of acetylcholine presumably due to cleavage of membrane protein SNAP‐25 (Blasi et al. 1993). It was reported for tracheal rings that botulinum neurotoxin A inhibits contractions initiated by electrical pulses but has only a minor effect on the contractions initiated by muscarinic agonists (Moffatt et al. 2004). Hence, treatment with BTX leaves muscarinic receptors in tracheal preparations free of endogenous stimulation by nerve release of acetylcholine, but fully susceptible for the stimulation by externally applied agonists or depolarization.

**Figure 2:**
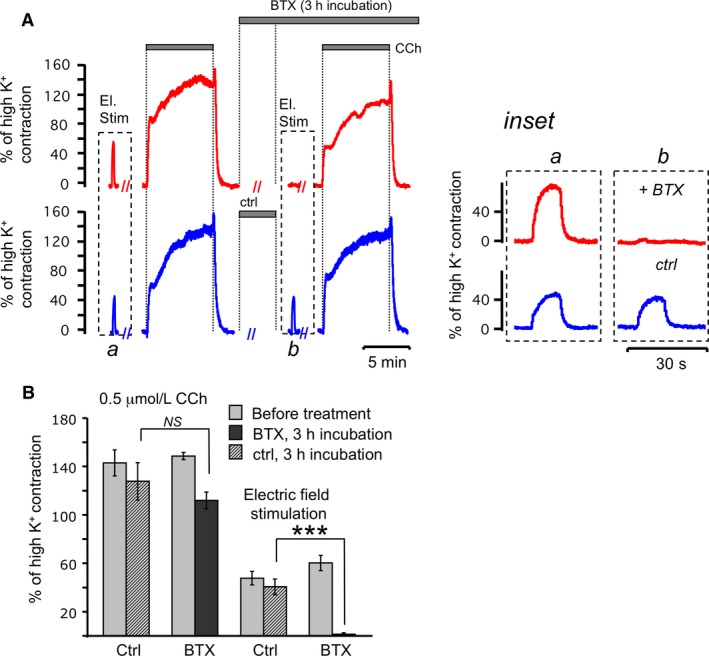
Botulinum toxin A (BTX) blocks ASM contraction activated by electric field stimulation but not by cholinergic agonist. (A) Representative traces of ASM contraction stimulated by CCh (0.5 *μ*mol/L) and electrical pulses (0.5 msec pulses, 30 Hz, 110 V/cm) before and after treatment with BTX (50 nmol/L) (red traces) in comparison to sham treatment (blue traces); *(inset)* magnified traces of ASM contraction stimulated by electrical pulses before and *(b)* after treatment with BTX. (B) Summarized data for A. Amplitude was estimated after contractions reached a plateau phase. Results are expressed as the means ± standard error of the mean. (***)*P* < 0.001, NS is nonsignificant (>0.05) For any bar *n* = 6.

We corroborated previous studies (Moffatt et al. 2004) by testing the effects of BTX on the contractions initiated by electrical field stimulation and cholinergic agonist (Fig. 2). Because BTX requires a sustained incubation time (3 h) to fully enter nerve endings, we included a no‐treatment time control (Fig. 2A, bottom). We found that contractions initiated by electrical field stimulation (Fig. 2A, dashed boxes, expanded in inset, 60 ± 6% of contraction initiated by high K^+^), presumably acting through cholinergic nerves, can be completely blocked after incubation of the tracheal preparations for 3 h with 50 nmol/L of BTX (1 ± 0.4% after BTX, *n* = 6). Nontreated trachea showed a reproducible response to a second electric field stimulation (2A and inset, bottom). These results indicate that electrical stimulation initiates contraction of ASM mainly due to acetylcholine release from nerve terminals. By contrast, BTX had only a small effect (148 ± 3% of high K^+^ contraction before vs. 111 ± 6% after treatment, *n* = 3) on contractions stimulated by exogenous application of 0.5 *μ*mol/L of cholinergic agonist carbachol (CCh). Control tracheas also showed some decrease in contractile force (142 ± 10% vs. 127 ± 15%, *n* = 6) after incubation for 3 h with an equivalent amount of vehicle (Fig. 2A, summarized in 2B).

**Figure 3:**
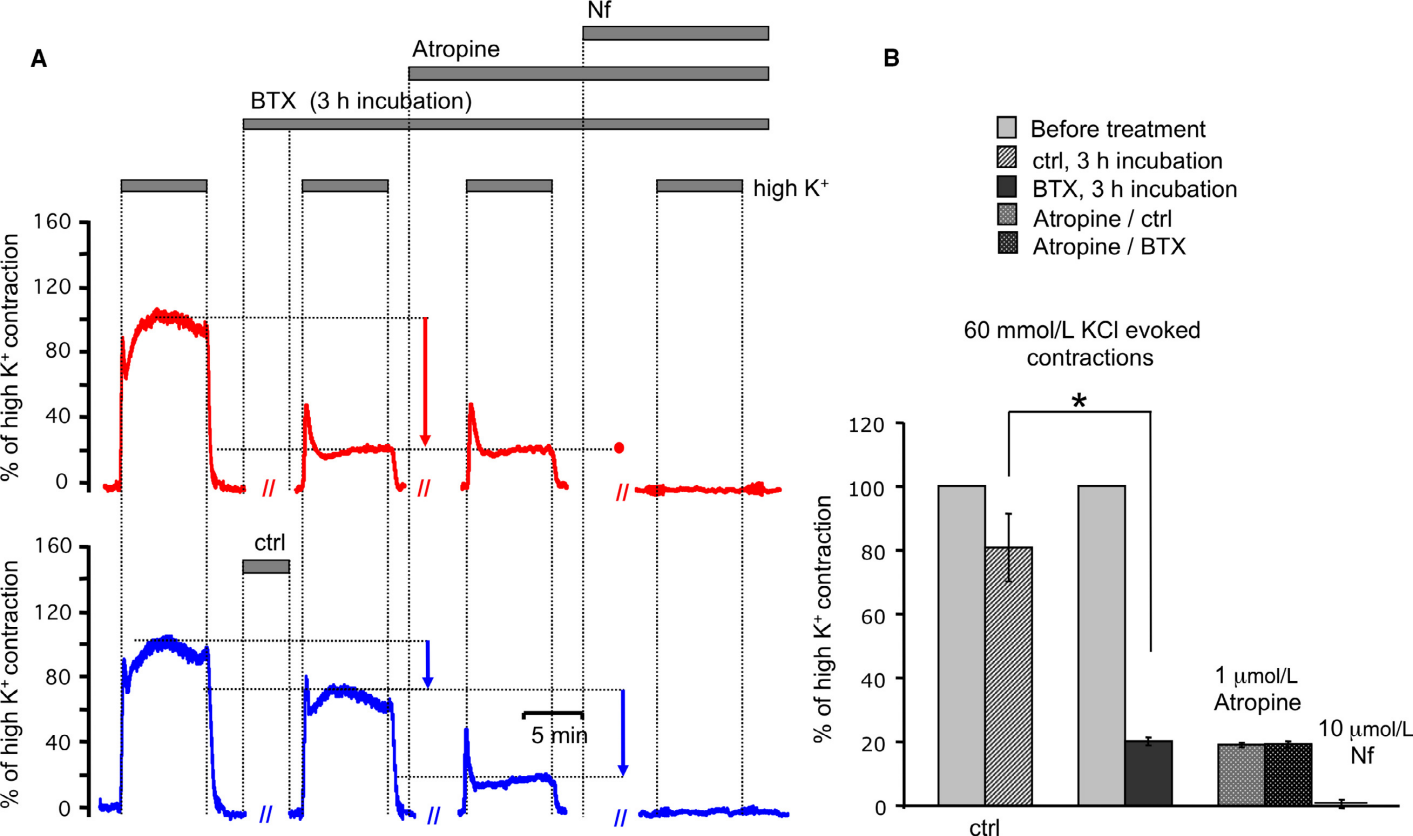
The majority of the steady‐state high K^+^ contraction is due to nerve‐evoked (BTX‐sensitive) activity. (A) Representative traces of ASM contraction in high K^+^ PSS before and after treatment with BTX (50 nmol/L) (red traces) in comparison to sham treatment (blue traces). Atropine (1 *μ*mol/L) has no additional effect after BTX treatment as opposed to control. In the presence of Atropine, Nifedipine (10 *μ*mol/L) blocks all remaining contractile activity in ASM regardless of pretreatment with BTX. (B) Summarized data for A. Amplitude was estimated after contractions reached a plateau phase. Results are expressed as the means ± standard error of the mean. (*)*P* < 0.05. For any bar *n* = 6.

We utilized BTX to exclude acetylcholine release from nerve endings and investigated high K^+^ depolarization‐mediated contractions. Tracheal preparations subjected to BTX (50 nmol/L, top Fig. 3A) and vehicle (bottom, Fig. 3A) were treated simultaneously. Contractions initiated by high K^+^ were measured before and after 3 h of incubation with BTX (Fig. 3A top) or vehicle (Fig. 3A bottom). Incubation with BTX significantly reduced the amplitude of contractions initiated by high K^+^ (Fig. 3A, summarized in 3). In the presence of BTX, high K^+^ contractile responses were dramatically reduced relative to pre‐BTX treatment, or the vehicle control (Fig. 3A, bottom). The BTX‐insensitive component had a prominent two‐phase appearance with the transient peak (53 ± 7% of high K^+^ contraction, *n* = 6) and stable plateau (20 ± 2% of high K^+^ contraction). This remaining BTX‐insensitive component (Fig. 3A, top) was accounted for by L‐type voltage‐gated calcium channels as it was insensitive to Atropine (1 *μ*mol/L), and eliminated by L‐type blocker Nifedipine (10 *μ*mol/L). In conclusion, high K^+^ contraction is mediated by two mechanisms. The majority of the sustained contraction is mediated by depolarization and release of acetylcholine from nerve endings resulting in a pharmacomechanical coupling. The remaining contraction is electromechanical coupling due to voltage‐dependent calcium channel activation, and calcium influx into ASM. We can also conclude that unliganded muscarinic receptors do not contribute to contraction since the remaining BTX‐insensitive contractile response is accounted for by Nifedipine‐sensitive, voltage‐dependent calcium channels. Based on these data, we find that contractions mediated through smooth muscle muscarinic receptors can be observed in high K^+^ solution by blocking neurotransmission with either BTX (Figs. 2 and 3), or 0 Ca^2+^ extracellular solution (Fig. 1). The remaining contractions mediated via postsynaptic voltage‐dependent calcium channels can be blocked with Nifedipine (Fig. 3).

**Figure 4:**
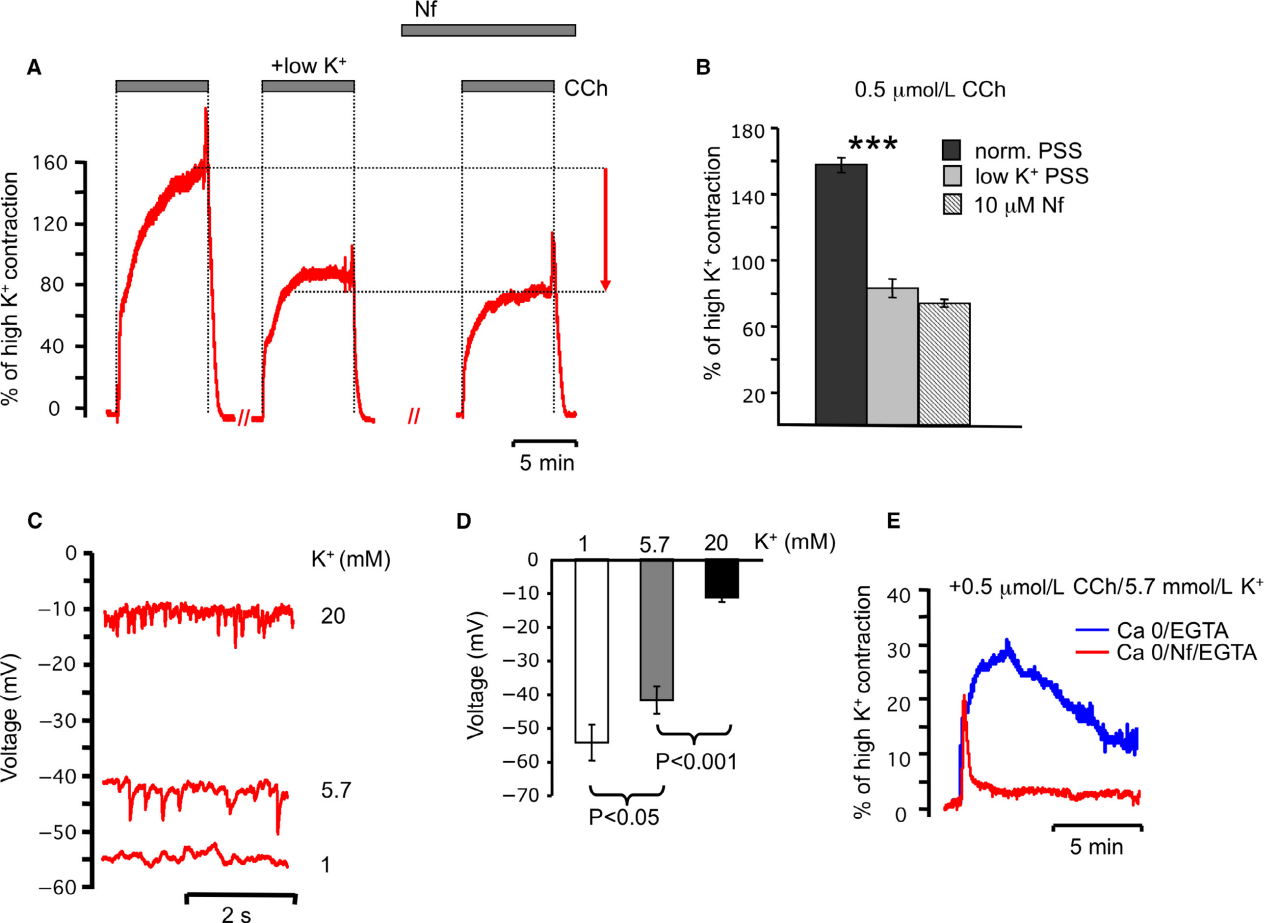
Low K^+^ PSS (1 mmol/L) hyperpolarize and high K^+^ PSS (20 mmol/L) depolarize membrane potential of ASM cells. (A) Representative traces of ASM contraction stimulated by CCh (0.5 *μ*mol/L) in the control conditions and low K^+^ PSS. Nifedipine (10 *μ*mol/L) does not have statistically significant effect on the CCh‐evoked contraction compared to tracheas incubated in low K^+^ PSS. (B) Summarized data for A. Amplitude was estimated after contractions reached a plateau phase. Results are expressed as the means ± standard error of the mean. For any bar *n* = 6. (C) Representative traces of resting potentials in ASM perfused with 1, 5.7, and 20 mmol/L K^+^ PSS. (D) Summarized data for C. Results are expressed as the means ± standard error of the mean. (E) Representative traces of ASM contractile response to cholinergic agonist in the 0 Ca^2+^/100 *μ*mol/L EGTA PSS with (red trace) and without (blue trace) Nifedipine (10 *μ*mol/L).

Before proceeding with study of voltage effects on muscarinic receptor signaling, we also investigated the various K^+^ solutions’ effect on ASM membrane potentials. We found that membrane potential in low potassium (1 mmol/L) PSS was hyperpolarized (Fig. 4C and D). Low K^+^ solution reduces contractility in response to cholinergic activation (Fig. 4 A and B), which likely occurs through hyperpolarization and reduced voltage‐gated Ca^2+^ channels activation since channel blocker Nifedipine (10 *μ*mol/L) blocks a very similar component of contractile force as low K^+^ solution (Fig. 4A, summarized in 4B). Thus, to study voltage sensitivity of muscarinic receptors, we concluded that solutions that allow voltage‐dependent Ca^2+^ influx should be avoided. As discussed above, 0 external calcium also avoids neurotransmitter release. Our protocol therefore used 0 Ca^2+^ PSS to study voltage sensitivity of muscarinic receptors upon cholinergic stimulation (CCh, 0.5 *μ*mol/L). To buffer any residual external Ca^2+^ ions, 100 *μ*mol/L of EGTA was added to the solution. Before application of CCh, organ baths (volume of 15 mL) were perfused for 1.5 min (67 mL/min) with 0 Ca^2+^ PSS to washout Ca^2+^ ions. To restore the Ca^2+^ content of SR after cholinergic stimulation in 0 Ca^2+^ solution, trachea preparations were perfused with normal PSS for 20 min between each CCh administration. Remarkably, we found that Nifedipine application also had an effect on the contractile response stimulated by CCh (0.5 *μ*mol/L) in 0 Ca^2+^ solution (Fig. 4E). In the presence of Nifedipine, contractile responses were reduced almost completely to a transient peak and a very shallow but measurable plateau. These responses had quite different kinetics in comparison to responses in 0 Ca^2+^ solutions that did not contain Nifedipine (Fig. 4E, plateau @ 5 min 29.3 ± 2.5 control vs. 3.5 ± 0.8 with Nifedipine). Nifedipine (10 *μ*mol/L) was therefore added to all solutions to prevent possible effects of direct coupling of L‐type Ca^2+^ channels to RyR1 (Du et al. 2006) or G_q_ proteins (del Valle‐Rodriguez et al. 2003). Solutions containing various concentrations of K^+^ ions (1, 5.7, 20, and 60 mmol/L) were used to control membrane potential of ASM plasma membranes.

### Physiological voltage changes do not affect muscarinic evoked contractions

**Figure 5:**
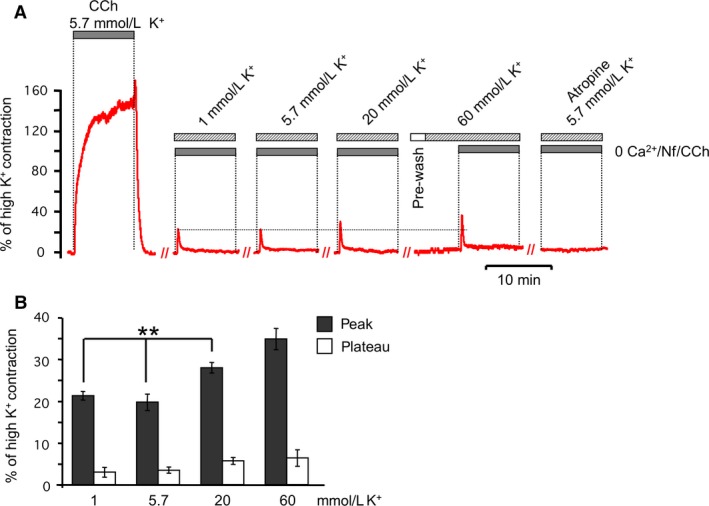
Voltage has a small influence on muscarinic receptor‐evoked contractions. (A) Representative traces of ASM contraction stimulated by CCh (0.5 *μ*mol/L) in extracellular solutions with different concentrations of K^+^ ions. Extracellular solutions contained 0 Ca^2+^ PSS in the presence of EGTA (100 *μ*mol/L) and Nifedipine (10 *μ*mol/L). (B) Summarized data for A. Amplitude was estimated at the peak of the contractile responses. Results are expressed as the means ± standard error of the mean. (**)*P* < 0.01. For any bar *n* = 5.

Figure 5A shows the contractile response that is due to isolated effects of muscarinic receptor activation, and at various voltages controlled by external potassium concentrations. The cholinergic‐evoked contractile response in normal K^+^ (Fig. 5A, 5.7 mmol/L K^+^) in the absence of calcium influx is small compared to contractions with calcium influx (Fig. 5A, CCh, 5.7 mmol/L K^+^). In contrast to calcium influx pathways that were highly sensitive to 1 mmol/L hyperpolarizing K^+^ solution (Fig. 4A), cholinergic‐evoked contractions in absence of calcium influx were unaffected by membrane hyperpolarization (Fig. 5A, 1 mmol/L K^+^). No differences in amplitudes of contractile responses were observed in 1 mmol/L K^+^ and 5.7 mmol/L K^+^ PSS (Fig. 5A, summarized in 5B), suggesting that cholinergic signaling is insensitive to physiological voltage changes.

To depolarize the membrane above the physiological range for smooth muscle, we evoked contractions in the presence of a 20 mmol/L K^+^ PSS solution. Voltage measurements in this solution indicated an average voltage of −12 ± 3 mV (*n* = 12, Fig. 4C and summarized in D), which is well above the average −44 ± 2 mV potential (*n* = 40, Fig. 4C and summarized in D) in normal PSS solution. We observed a small, but significant potentiation of transient peak by the depolarization with 20 mmol/L K^+^ (from 19.7 ± 2% to 28 ± 1.2%, *P* ≤ 0.01, *n* = 5, Fig. 5A and summarized in 5B). Cholinergic stimulation in the presence of 60 mmol/L K^+^ initiated transient contractile response with the tendency to be larger (35 ± 6, *n* = 5) than response in 20 mmol/L K^+^ (Fig. 5A), although this was not statistically significant increase. Application of Atropine abolished all contractile activity in the 0 Ca^2+^ PSS (Fig. 5A) confirming the role of muscarinic receptors. In summary, changes in voltages from normal PSS to hyperpolarizing solutions appear to have little effect on muscarinic receptor‐evoked contractions. However, depolarizing voltage increases, as provided by 20 or 60 mmol/L K^+^ solutions, did increase muscarinic‐evoked contractions.

## Discussion

The focus of this study was to evaluate the physiological effects of voltage on muscarinic receptor‐evoked ASM contractility. Our data suggest that (1) effect of voltage sensitivity of muscarinic receptors on ASM contraction becomes significant only at plasma membrane potentials that are beyond physiological ranges and (2) depolarization of plasma membrane alone cannot activate contractions through muscarinic receptors; ligand binding is also required.

### Major signaling cascades evoked by high K^+^ depolarization

High K^+^ contractions are often used in smooth muscle contractility studies to normalize contractile responses to the mass or health of a tissue preparation (Herlihy et al. 1032; Semenov et al. 2012). It was generally assumed that high K^+^ solution mediates contraction through smooth muscle depolarization and activation of voltage‐gated calcium channels. In isolating conditions to evaluate voltage effects on muscarinic receptors, we can infer that a larger component of the contraction was due to high K^+^ depolarization of parasympathetic nerves that release acetylcholine onto apposing airway smooth muscle cells. This causes activation of muscarinic receptor signaling and calcium release, and also voltage‐dependent, calcium influx pathways to cause muscle contraction. Thus, contraction of ASM in high K^+^ solution has two major pharmacologically sensitive parts: first – a large atropine‐sensitive component due to indirect effects of depolarization on cholinergic nerves; and second – a smaller nifedipine‐sensitive component due to direct effects of depolarization on voltage‐dependent ASM channels. The existence of the two pathways explains why neither nifedipine nor atropine could abolish contraction, and only application of both could completely prevent the contractile response to high K^+^.

### Zero Ca2^+^ external solution and Nifedipine isolates contractile effects of muscarinic receptors

An important aspect of these studies was to identify conditions for isometric contractions that are due only to smooth muscle muscarinic receptors. Ultimately, we found that 0 calcium external solution suited this purpose. The 0 external calcium solution prevents high K^+^ depolarization‐induced acetylcholine release from nerve endings. As well, 0 calcium external solution with added Nifedipine prevents voltage‐dependent calcium influx in the smooth muscle. Depolarization has also been shown to activate a Rho and Rho‐activated kinase‐dependent contraction, which is blocked by Nifedipine (Liu et al. 2005). It is likely that some calcium‐dependent signaling cascades, downstream of muscarinic receptor activation, are also prevented using 0 external calcium external solution. Conceptually, this would include store‐operated calcium influx that follows muscarinic‐evoked calcium release (Ay et al. 2004), and second messenger–activated calcium permeable channels such as TrpM and TrpC channels (Gosling et al. 2005). The remaining calcium signaling that is downstream of Gq‐coupled M3 receptor activation likely underlies the contraction due to high K^+^ depolarization and muscarinic receptor activation that is the focus of this study. This likely includes IP3 receptor‐mediated calcium release, and calcium‐induced (ryanodine receptor) calcium release. As well, Rho‐dependent contractions can also be mediated by calcium release mechanisms (Liu et al. 2005).

Although this study carefully investigated the influence of cholinergic nerves which play a predominant role, it is worth noting that other cell types, such as airway epithelial cells (Vanhoutte 2013) and sympathetic nerves (van Nieuwstadt et al. 1994), can release factors in response to chemical depolarization that influence contractility of tracheal smooth muscle cells. These are factors released from tissue such as prostaglandins and nitric oxide (Ruan et al. 2011; Kloesch et al. 2016; Blatter 2017), and autonomic neurotransmitters such as norepinephrine (Garssen et al. 1990) that affect ASM. Under conditions where we attempted to isolate depolarization‐mediated effects, then 0 calcium external solution would be expected to prevent depolarization‐induced secretion of these factors or sympathetic neurotransmitter release (Atlas 2001). Indeed, the ability of the 0 calcium, nifedipine external solution to isolate effects due to muscarinic receptors was corroborated by the observation that muscarinic antagonist atropine completely reversed the contractions (Fig. 5). However, future studies are needed to conclusively exclude the contributions of other tissues on chemical depolarization‐induced contractions.

### Depolarization of unliganded muscarinic receptors do not affect ASM contractility

Our experiments that used high K^+^ to depolarize muscle and BTX to prevent acetycholine release from nerve endings (Fig. 3) allowed us to determine if voltage‐activated, unliganded muscarinic receptors affect contraction. Under these circumstances, we saw no atropine‐sensitive component of contraction, which suggest that unliganded muscarinic receptors have no role in contractility, despite depolarization. Similar to our findings, others have shown that ligand binding is required for voltage‐dependent effects on muscarinic receptors (Rinne et al. 2015). As expected, depolarization‐induced contractions, in the absence of acetylcholine release (BTX), were solely due to L‐type voltage‐gated calcium channels, as these contractions were fully blocked by Nifedipine.

### Physiological voltage increases do not affect muscarinic‐evoked contractions

A key finding was that the isolated muscarinic‐evoked contractions (Fig. 5A) were insensitive to hyperpolarizing solution, suggesting that muscarinic receptors are not affected by physiological voltages. The explanation may be that depolarization of ASM plasma membrane upon cholinergic stimulation is considered to be relatively moderate (Janssen 2002). Our own studies indicate that application of half‐maximal concentrations of CCh results only in 5–7 mV depolarization of ASM plasma membrane from average resting voltages of −44 mV (Semenov et al. 2011; Evseev et al. 2013). Such depolarization falls within a voltage window that recruits nifedipine‐sensitive calcium channels to contractions (Fleischmann et al. 1994), and this effect was reversed by hyperpolarizing solution (Fig. 4). Nevertheless, depolarization with 20 mmol/L K^+^ PSS, that depolarizes plasma membrane to nonphysiological voltages, from ‐44 mV to −12 mV (Fig. 5), did yield measurable effects on muscarinic receptor‐mediated contraction. In past studies of voltage effects on ASM muscarinic receptor‐evoked contractions, potassium solutions were used that cause depolarizing voltages changes (Liu et al. 2009), consistent with the effect observed with 20 and 60 mmol/L K^+^ solutions (Fig. 5). Previous studies have shown that Ca^2+^ release triggered by cholinergic stimulation in coronary myocytes can be potentiated by significant (to 0 mV) depolarization (Ganitkevich and Isenberg 1993). The mechanism is ascribed to a depolarization‐induced conformational change in the GPCR domain that couples to the G protein. As a result, the likelihood of the receptor's coupling to the G protein increases, and so is the affinity of the receptor toward the agonist (Ben‐Chaim et al. 2006).

## Conclusion

Our goal was to isolate muscarinic receptor signaling in an intact ASM tissue, and investigate the physiological consequences of voltage sensitivity on contractile responses. Our results suggest that while large chemical depolarizations do increase contractility, the physiological range of membrane potentials of ASM has minimal if any effect on the muscarinic‐evoked contractile response of ASM. However, muscarinic receptors do occur in phasic smooth muscle tissues such as bladder and gut smooth muscle that undergo larger depolarization (Young 2007; van Helden et al. 2010). Studies in those tissue may reveal a larger role for voltage activation of muscarinic receptors.

## Conflict of Interest

No conflicts of interest, financial or otherwise, are declared by the authors.
